# Brain *Versus* Blood: A Systematic Review on the Concordance Between Peripheral and Central Kynurenine Pathway Measures in Psychiatric Disorders

**DOI:** 10.3389/fimmu.2021.716980

**Published:** 2021-09-23

**Authors:** Katrien Skorobogatov, Livia De Picker, Robert Verkerk, Violette Coppens, Marion Leboyer, Norbert Müller, Manuel Morrens

**Affiliations:** ^1^ Faculty of Medicine and Health Sciences, Collaborative Antwerp Psychiatric Research Institute (CAPRI), University of Antwerp, Antwerp, Belgium; ^2^ Scientific Initiative of Neuropsychiatric and Psychopharmacological Studies (SINAPS), University Psychiatric Centre Duffel, Duffel, Belgium; ^3^ Laboratory of Medical Biochemistry, University of Antwerp, Antwerp, Belgium; ^4^ INSERM U955, Equipe Psychiatrie Translationnelle, Créteil, France; ^5^ Fondation FondaMental - Hôpital Albert Chenevier - Pôle Psychiatrie, Créteil, France; ^6^ AP-HP, Hôpitaux Universitaires Henri Mondor, DHU Pepsy, Pôle de Psychiatrie et d’Addictologie, Créteil, France; ^7^ Université Paris Est Créteil, Faculté de Médecine, Creteil, France; ^8^ Department of Psychiatry and Psychotherapy, Ludwig-Maximilians-University, München, Germany

**Keywords:** kynurenine, blood-brain barrier, immune, tryptophan, psychiatry, inflammation, CSF

## Abstract

**Objective:**

Disturbances in the kynurenine pathway have been implicated in the pathophysiology of psychotic and mood disorders, as well as several other psychiatric illnesses. It remains uncertain however to what extent metabolite levels detectable in plasma or serum reflect brain kynurenine metabolism and other disease-specific pathophysiological changes. The primary objective of this systematic review was to investigate the concordance between peripheral and central (CSF or brain tissue) kynurenine metabolites. As secondary aims we describe their correlation with illness course, treatment response, and neuroanatomical abnormalities in psychiatric diseases.

**Methods:**

We performed a systematic literature search until February 2021 in PubMed. We included 27 original research articles describing a correlation between peripheral and central kynurenine metabolite measures in preclinical studies and human samples from patients suffering from neuropsychiatric disorders and other conditions. We also included 32 articles reporting associations between peripheral KP markers and symptom severity, CNS pathology or treatment response in schizophrenia, bipolar disorder or major depressive disorder.

**Results:**

For kynurenine and 3-hydroxykynurenine, moderate to strong concordance was found between peripheral and central concentrations not only in psychiatric disorders, but also in other (patho)physiological conditions. Despite discordant findings for other metabolites (mainly tryptophan and kynurenic acid), blood metabolite levels were associated with clinical symptoms and treatment response in psychiatric patients, as well as with observed neuroanatomical abnormalities and glial activity.

**Conclusion:**

Only kynurenine and 3-hydroxykynurenine demonstrated a consistent and reliable concordance between peripheral and central measures. Evidence from psychiatric studies on kynurenine pathway concordance is scarce, and more research is needed to determine the validity of peripheral kynurenine metabolite assessment as proxy markers for CNS processes. Peripheral kynurenine and 3-hydroxykynurenine may nonetheless represent valuable predictive and prognostic biomarker candidates for psychiatric disorders.

## 1 Introduction

Immune dysregulation plays an important role in the pathophysiology of several psychiatric disorders. Mood and psychotic disorders exhibit peripheral and central immune abnormalities, such as increased peripheral pro-inflammatory cytokine levels (1–3) and up- or downregulated central nervous system (CNS) glial responses (4–7). Immune mechanisms are further known to modulate psychiatric symptom development and illness course. Specifically, inflammation-induced depressive symptomatology has been observed in healthy volunteers and patients recently remitted from major depression (8, 9), while add-on anti-inflammatory drugs improve residual symptoms in patients with major depressive disorder (MDD) and psychotic disorders. This effect is particularly observed if patients present with a basally increased peripheral pro-inflammatory cytokine profile (10, 11).

For over half a century, disruption of the kynurenine pathway (KP) has been proposed as a mechanistic link between immune disturbances and psychiatric pathology and symptomatology (12, 13). Since the early 1990s, increased efforts and better analytical methods have further disclosed the role of tryptophan (TRP), kynurenine (KYN) and their downstream metabolites in psychotic and mood disorders. Two meta-analyses (14–16) have demonstrated that a.o. peripheral tryptophan, kynurenine and kynurenic acid levels are at least partially downregulated in mood and psychotic disorders, whereas the limited number of studies focusing on cerebrospinal fluid (CSF) and brain tissue demonstrate unaltered or even increased KP metabolite concentrations (especially kynurenic acid) in these disorders (17–20). Most clinical studies to date quantified kynurenine metabolite concentrations in peripheral blood. Nonetheless, fundamental knowledge about the interrelation between kynurenine metabolites in CNS and peripheral blood and, importantly, their bidirectional transport across the blood-brain barrier remains incomplete. Peripheral KP metabolite quantifications may not represent concentrations in CNS tissue, as evidenced by divergent research results. Consequently, the validity of peripheral kynurenine metabolite assessment as biomarkers for human neuropsychiatric illnesses has been questioned (14, 21, 22).

### 1.1 Overview of the Kynurenine Pathway

**Figure 1:**
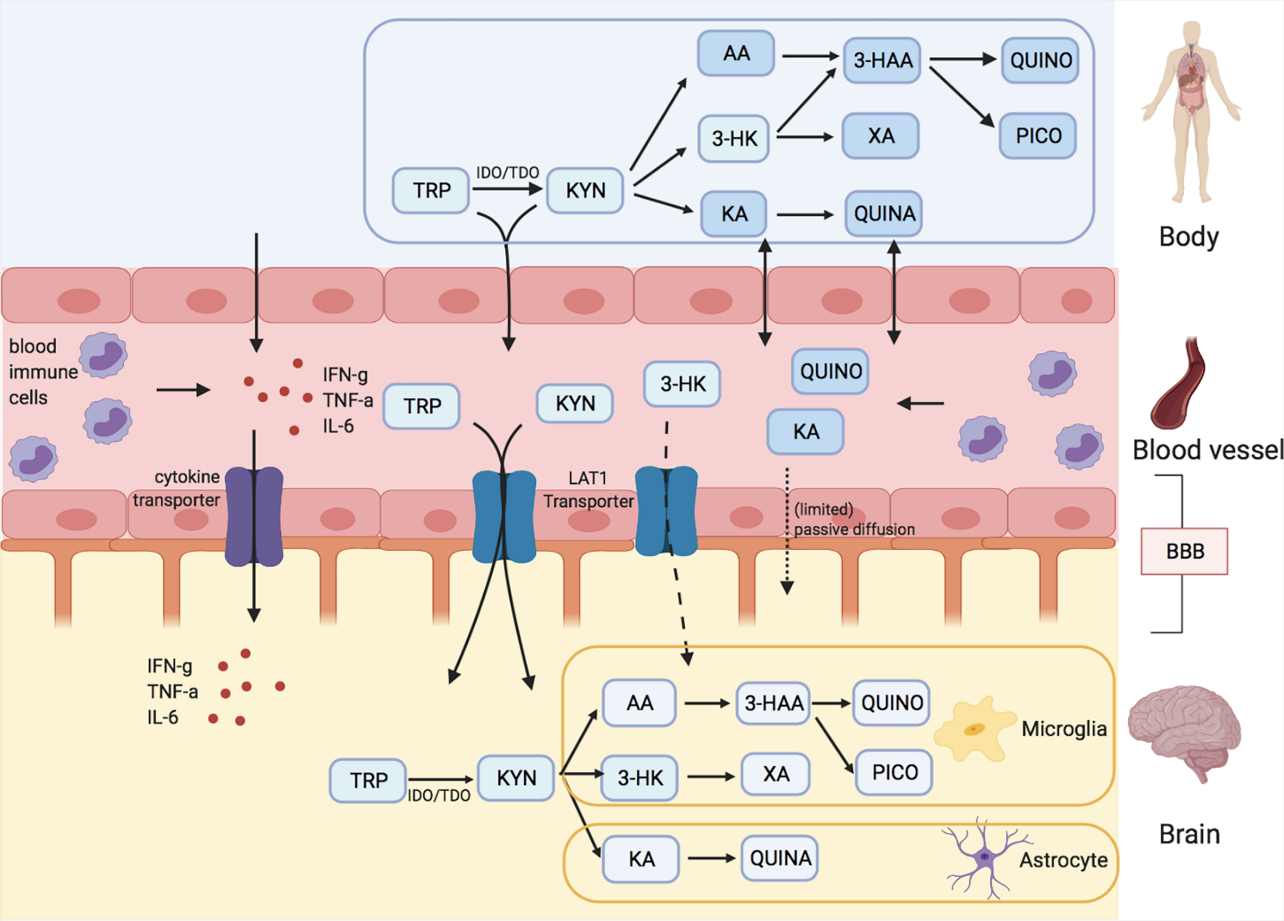
Kynurenine metabolites and the blood brain barrier (BBB). Tryptophan (TRP) and kynurenine (KYN), and to a lesser degree 3-hydrox kynurenine (3-HK) are actively transported into the brain over LAT1 transporters. Downstream metabolites of the kynurenine pathway (KP), like quinolinic acid (QUINO) and kynurenic acid (KA), cannot make use of these transporters, but (probably limited) passive diffusion of these metabolites over the BBB is possible. Anthranilic acid and 3-hydroxy anthranilic acid (not shown in figure) may equally pass the blood brain barrier through passive diffusion, much like QUINO. In the brain, microglia are responsible for the production of metabolites 3-HK and QUINO, whereas astrocytes produce KA. Peripheral production of these KP metabolites is done by blood immune cells, such as blood monocytes (PBMC) and other organs, including liver and kidney. The gut microbiome, which plays a role in psychiatric illness through the gut-brain axis, also affects KP metabolization.

Tryptophan (TRP) is an essential amino acid mainly known as the precursor of serotonin (5-HT) and melatonin. The first, rate-limiting, step of the pathway is the conversion of TRP to KYN by the enzymes indoleamine 2,3-dioxygenase (IDO) and tryptophan 2,3-dioxygenase (TDO) (**Figure 1**). TDO, which is mainly found in the liver and also in the brain (23–26), metabolizes 95% of whole-body TRP into KYN, of which the liver contributes 90%. Under normal physiological conditions, TDO in liver tissue will consume most of diet-derived TRP, and as such is the main source of KYN throughout the body (27). TDO is considered a housekeeping enzyme: excess TRP is diverted to the Krebs cycle to generate energy (26). The enzyme is induced by glucocorticoids to fulfill energy needs under stressful conditions and is thus activated by psychophysiological stress by cortisol release (28). Moreover, TDO is inhibited by a reduction in nicotinamide, activated by heme and stabilized by TRP (26).

Activity of IDO is low in non-pathological conditions but, unlike TDO, can be downregulated by anti-inflammatory cytokines (29) and upregulated by pro-inflammatory cytokines [mainly interferon gamma (IFN-g), but also tumor necrosis factor alpha (TNF-a)] (30) and psychological stress (31).

In the brain, downstream metabolization of KYN occurs through divergent routes in microglia and astrocytes. Kynurenine 3-monooxygenase (KMO), only active in cerebral microglia, metabolizes KYN into 3-hydroxykynurenine (3-HK), an N-methyl D-aspartate (NDMA) receptor agonist (32). Metabolites further downstream from 3-HK include quinolinic acid (QUINO), another NMDA-receptor agonist, and picolinic acid (PICO), which in contrast antagonizes the NMDA-receptor. It is assumed that additional microglial QUINO can be produced in parallel *via* catabolization of KYN to anthranilic acid (AA), although this was not supported by Giorgini et al. as QUINO levels were almost non-existent in KMO deficient mice (AA) (33). Quinolinic acid phosphoribosyltransferase (QPRT) further degrades QUINO to niacin, a form of vitamin B3.

In astrocytes, KYN is metabolized by kynurenine aminotransferases (KAT) to kynurenic acid (KA), another NMDA-receptor antagonist. However, a portion of this astrocyte produced KYN will fuel macrophages and microglia to produce QUINO (34). KA is considered a neuroprotective metabolite due to its antagonism effect on the excitatory NMDA receptor. However, abnormally elevated KA has been put forward as the mechanism causing glutamate hypofunction by sustained NMDA receptor antagonism, which can lead to psychotic symptoms and cognitive and social impairments in MDD and SCZ (35–37).

In the periphery, catabolization through KMO and KAT both occurs albeit at different rates depending on multiple factors such as the relative abundance of the enzymes in specific tissues, substrate concentration and affinity, pH, bioavailability of cofactors, cosubstrates and competing substrates (26). As the majority of studies investigating these enzymes are performed *in vivo*, these factors are often not taken into account resulting in a simplification of the actual enzyme physiology (38). KMO is mostly present in liver, kidney, macrophages and monocytes (39), while KAT is active in liver, kidney, placenta, heart and macrophages (40). Pro-inflammatory cytokines like IFN-g also have a strong stimulating effect on KMO, in the brain as well as in the periphery.

### 1.2 Study Objectives

As the primary objective of this systematic review, we will investigate the correlation coefficients between peripheral and central kynurenine metabolite concentrations in preclinical research and in human samples of varying origins. As secondary objectives, associations between peripheral KP measures and (endo)phenotypic measures (symptom severity, treatment response and CNS abnormalities) in psychiatric illness will be described to appraise the value of KP metabolites as prognostic and predictive biomarkers. In order to provide a better understanding of the factors influencing central and peripheral KP metabolites, we will discuss the (patho)physiological impact on KP bioavailability and blood-brain-barrier (BBB) transport in healthy and immune-activated physiological states.

## 2 Methodology

We performed a pubmed-based literature systematic search (January 1968 - February 2021) using the following search string: ((tryptophan OR kynuren* OR “quinolinic” OR “xanthurenic acid” OR “anthranilic acid”) AND (“serum” OR “plasma” OR “blood”) AND (“brain tissue” OR “BBB” OR “blood brain barrier” OR “blood-brain-barrier” OR “CSF” OR “cerebrospinal fluid” OR “postmortem” OR “post-mortem” OR “MRI” OR “fMRI” OR “PET” OR “DTI”) NOT (review [Publication Type])). Eligible papers were extracted from the PubMed database using the following inclusion criteria: 1) English language articles published in peer-reviewed journals, 2) Human studies including patients with a major psychiatric disorder reporting correlation coefficients between peripheral and central KP metabolites or association measures between at least one peripheral KP metabolite and symptom severity or brain imaging disturbances or treatment outcome, or 3) Human studies including healthy controls or non-psychiatric patients reporting correlation coefficients between peripheral and central KP metabolites, or 4) *In-vivo* or postmortem assessment of at least one KP metabolite peripherally and centrally.

Two authors independently performed the literature search (M.M., K.S.). This search strategy yielded 1078 records that, with 19 additional records found through cross-referencing resulted in a total of 1097 records that were screened based on title and abstract. After exclusion of 868 irrelevant records, full articles were evaluated of 229 papers, ultimately leading to 59 papers that were included in the systematic review.

We included 27 original research articles describing a correlation between peripheral and central kynurenine metabolite measures in preclinical studies and human samples from healthy controls and patients suffering from neuropsychiatric disorders and other conditions. Correlation coefficients of peripheral-central KP metabolites along with the p-values, sample type, sample size and pathology type were extracted from the articles. The strength of concordance between peripheral and central KP measures was evaluated as a function of correlation measures, i.e. discordance = r <.20; weak concordance = .20 ≤ r ≤ -.39; moderate concordance = .40 ≤ r ≤ -.59 and strong concordance = r ≥.60 (41, 42).

We also included 32 articles reporting associations between peripheral KP markers and symptom severity, CNS pathology or treatment response in schizophrenia (SCZ), bipolar disorder (BD) or major depressive disorder (MDD).

**Figure 2:**
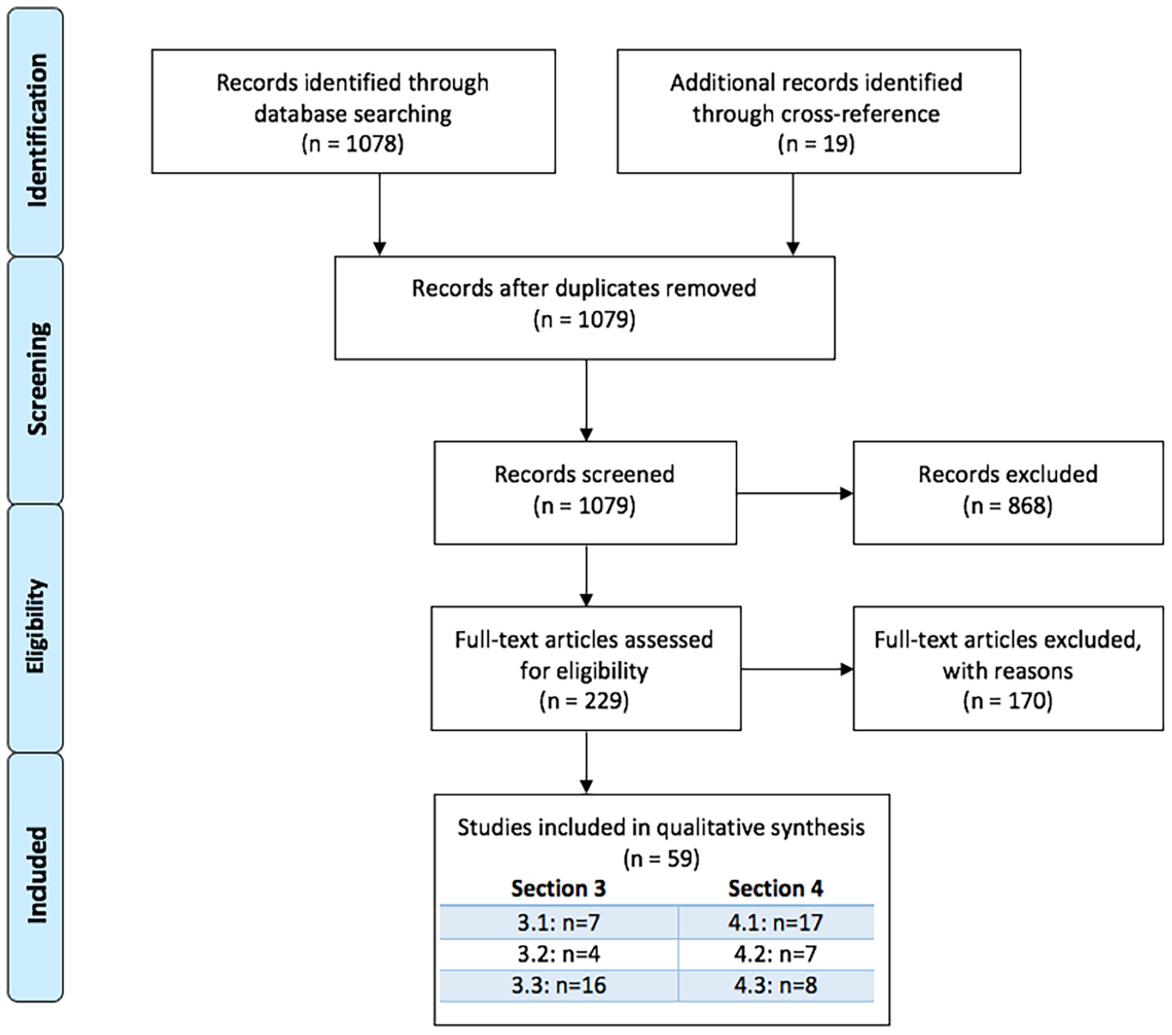
PRISMA flowchart.

See **Figure 2** for PRISMA Flow Diagram [based on (43)].

## 3 Concordance of Peripheral and Central KP Metabolite Assessments

(**Table 1**) provides an overview of the included studies investigating correlations between peripheral and central kynurenine metabolite concentrations in both human and preclinical research. Since only a handful of studies (n=4) reported on these correlations in psychiatric populations, we additionally listed findings in healthy subjects and non-psychiatric diseases (n=14). It should be noted that the available psychiatric studies only concern depressed and bipolar patients, as until date no study has scrutinized this association in schizophrenic patients.

**Table 1:** Overview of studies investigating correlations between blood-based and CSF kynurenine pathway metabolites.

Study	Animal	Sample type	Sample size	Metabolite	r-value	p-value
**PRECLINICAL RESEARCH**						
*Tryptophan*					Conflicting results	
Crandall et al. (1983) (44)	Normal and diabetic rats	serum//brain	n=36	TRP (total)	.53	<.001
Sarna et al. (1982) (45)	Rats	plasma/brain	n=23	TRP (total)	-.69	NS
Sarna et al. (1982) (45)	Rats	plasma/brain	n=23	TRP (free)	.29	NS
Gabriel Manjarrez et al. (2001) (46)	Rats undernourished in utero	plasma/brain (auditory cortex)	n=30	TRP (free)	.95	<.05
Yokogoshi et al. (1987) (47)	Rats receiving amino acid supplementation	plasma/brain	n=54	TRP (not specified)	.95	<.001
Verdonk et al. (2019) (48)	Mice receiving an immune challenge	plasma/brain	n=60	TRP (not specified)	-.21	NS
*Kynurenine*					Strong concordance	
Gregoire et al. (2008) (49)	Monkeys	serum/CSF	n=8	KYN	.60	.011
Verdonk et al. (2019) (48)	Mice receiving an immune challenge	plasma/brain	n=60	KYN	.86	<.0001
*Kynurenic acid*					Conflicting results	
Gregoire et al. (2008) (49)	Monkeys	serum/CSF	n=8	KA	.42	NS
*Quinolinic acid*					Strong concordance	
Saito et al. (1993) (50)	Immune stimulated gerbils	plasma/CSF	n=5	QUINO	.97	<.01
Verdonk et al. (2019) (48)	Immune stimulated mice	plasma/brain	n=60-75	QUINO	.71	<.0001
*3-hydroxykynurenine*					Moderate-strong concordance	
Verdonk et al. (2019) (48)	Mice receiving an immune challenge	plasma/brain	n=60-75	3-HK	.72	<.0001
**Study**	**Pathology**	**Sample type**	**Sample size**	**Metabolite**	**r-value**	**p-value**
**HUMAN RESEARCH**						
**Psychiatric diseases**						
*Tryptophan*					Discordance	
Moreno et al. (2010) (51)	Remitted MDD	plasma/CSF	n=21	TRP (total)	.15	NS
Hestad et al. (2017) (17)	MDD	serum/CSF	n=75 (MDD n=44)	TRP (not specified)	.21	NS
Haroon et al. (2020) (52)	MDD	plasma/CSF	n=72	TRP (not specified)	N/A	NS
*Kynurenine*					Strong concordance	
Hestad et al. (2017) (17)	MDD	serum/CSF	n=75 (MDD n=44)	KYN	.61	<.001
Haroon et al. (2020) (52)	MDD	plasma/CSF	n=72	KYN	.60	<.0001
*Kynurenic acid*					Discordance	
Sellgren et al. (2019) (20)	BD	plasma/CSF	BD n=163	KA	.15	NS
Haroon etal. (52)	MDD	plasma/CSF	n=72	KA	N/A	NS
*Quinolinic acid*					Moderate concordance	
Haroon et al. (2020) (52)	MDD	plasma/CSF	n=72	QUINO	.55	<.0001
*Anthranilic acid*					Moderate concordance	
Haroon et al. (2020) (52)	MDD	plasma/CSF	n=72	AA	.47	<.0001
**Nonpsychiatric diseases**						
*Tryptophan*					Total TRP: 3 of 5 studies show discordant results Free TRP: 3 of 6 studies show discordant results Not specified TRP: 3 of 4 studies show discordant results	
Young et al. (1975) (53)	Healthy volunteers	Serum/CSF	n=29	TRP (total)	.47	<.05
Sullivan et al. (1978) (54)	Healthy volunteers	Plasma/CSF	n=13	TRP (total)	.25	NS
Kruse et al. (1985) (55)	Healthy volunteers	Serum/CSF	n=44	TRP (total)	.30	.01
Young et al. (1976) (56)	59 year old man after neurosurgery (ventricular drain)	Serum/ventricular CSF	n=1 (case 1)	TRP (total)	.28	NS
Young et al. (1976) (56)	21 year old man with acute meningitis (ventricular drain)	Serum/ventricular CSF	n=1 (case 2)	TRP (total)	.26	NS
Sullivan et al. (1978) (54)	Uraemic patients	Plasma/CSF	n=14	TRP (total)	.22	NS
Gillman et al. (1980) (57)	Psychosurgery patients	Plasma/brain tissue	n=5	TRP (total)	.58	NS
Young et al. (1975) (53)	Healthy volunteers	Serum/CSF	n=29	TRP (free)	.02	NS
Sullivan et al. (1978) (54)	Healthy volunteers	Plasma/CSF	n=10	TRP (free)	.22	NS
Young et al. (1976) (56)	59 year old man after neurosurgery (ventricular drain)	Serum/ventricular CSF	n=1 (case 1)	TRP (free)	.57	<.05
Young et al. (1976) (56)	21 year old man with acute meningitis (ventricular drain)	Serum/ventricular CSF	n=1 (case 2)	TRP (free)	.76	<.05
Gillman et al. (1980) (57)	Psychosurgery patients	Plasma/brain tissue	n=5	TRP (free)	.97	<.01
Curzon et al. (1980) (58)	Psychosurgery patients	Plasma/CSF	n=19	TRP (free)	.44	.02
Sullivan et al. (1978) (54)	Uraemic patients	Plasma/CSF	n=12	TRP (free)	.57	NS
Cangiano et al. (1990) (59)	Cancer patients and healthy volunteers	Plasma/CSF	n=28	TRP (free)	.57	<.01
Sarrias et al. (1990) (60)	healthy volunteers	plasma/CSF	n=35	TRP (not specified)	.34	NS
Isung et al. (2021) (61)	Healthy subjects	plasma/CSF	n=27	TRP (not specified)	.37	<.025
Heyes et al. (1992) (62)	HIV	serum/CSF	n=79	TRP (not specified)	.21	NS
Raison et al. (2010) (63)	Hepatitis	plasma/CSF	n=27	TRP (not specified)	.14	NS
*Kynurenine*					Moderate-strong concordance	
Isung et al. (2021) (61)	Healthy subjects	plasma/CSF	n=27	KYN	.27	NS
Heyes et al. (1992) (62)	HIV	serum/CSF	n=79	KYN	.65	<.0001
Raison et al. (2010) (63)	Hepatitis	plasma/CSF	n=27	KYN	.53	<.01
Havelund et al. (2017) (64)	Parkinson	plasma/CSF	n=26	KYN	.46	.03
Jacobs et al. (2019) (65)	AD	plasma/CSF	n=38 (AD n=20)	KYN	.70	<.001
*Kynurenic acid*					Conflicting results: 2 of 3 studies shown discordance	
Sellgren et al. (2019) (20)	Healthy volunteers	plasma/CSF	n=113	KA	-.02	NS
Havelund et al. (2017) (64)	Parkinson	plasma/CSF	n=26	KA	N/A	NS
Isung et al. (2021) (61)	Healthy subjects	plasma/CSF	n=27	KA	.51	<.01
*3-hydroxykynurenine*					Moderate concordance	
Havelund et al. (2017) (64)	Parkinson	plasma/CSF	n=26	3-HK	.51	.02
Jacobs et al. (2019) (65)	AD	plasma/CSF	n=38 (AD n=20)	3-HK	.33	.044
*Quinolinic acid*					Moderate-strong concordance in patients	
Isung et al. (2021) (61)	Healthy subjects	plasma/CSF	n=27	QUINO	.02	NS
Heyes et al. (1992) (62)	HIV	serum/CSF	n=111	QUINO	.43	<.0001
Valle et al. (2004) (66)	HIV	plasma/CSF	n=62	QUINO	.57	<.0001
Raison et al. (2010) (63)	Hepatitis	plasma/CSF	n=27	QUINO	.72	<.001
*Anthranilic acid*					Strong concordance	
Jacobs et al. (2019) (65)	AD	plasma/CSF	n=38 (AD n=20)	AA	.63	<.001
*Picolinic acid*					Moderate-strong concordance	
Isung et al. (2021) (61)	Healthy subjects	plasma/CSF	n=27	PICO	.93	<.0001
Jacobs et al. (2019) (65)	AD	plasma/CSF	n=38 (AD n=20)	PICO	.54	<.001

This table presents a summary of the concordance between peripheral and central kynurenine metabolites in preclinical, human psychiatric and non-psychiatric studies, sorted by metabolite. The correlations coefficients and its significance are represented as r-values and p-values respectively.

MDD, major depressive disorder; BD, bipolar disorder; AD, Alzheimer’s disease; HIV, human immunodeficiency virus; CSF, cerebrospinal fluid; TRP, tryptophan; KYN, kynurenine; KA, kynurenic acid; 3-HK, 3 hydroxykynurenine; QUINO, quinolinic acid; AA, anthranilic acid; PICO, picolinic acid; NS, non-significant.

### 3.1 Preclinical Findings

Overall, animal studies showed divergent results on peripheral-central TRP correlations (see **Table 1**) (44–48). In immune challenged mice (i.e. lipopolysaccharide) (48) plasma and brain parenchyma KYN levels strongly correlated (r=.86; p<.001), in contrast to TRP levels (r=-.21). Similarly, 3-HK displayed very good inter-tissue correlations (r=.72; p>.001). Animal studies (rats, mice, rabbits) using (supraphysiological) stimulation of the immune system showed very high between-tissue correlations for QUINO and KA (r>.70) in plasma, CSF and brain tissue (48, 50). When directly comparing CSF and serum of monkeys receiving a kynurenine 3-hydroxylase inhibitor, both kynurenine and KA correlated between CSF and serum (r=0.60 and 0.43, respectively) (49).

In amino acid supplemented rats, a significant increase in brain TRP concentrations was observed, accompanied by strong plasma/brain correlations (47). Goeden (67) demonstrated that administration of kynurenine to pregnant female rats leads to very comparable increases of KYN (9-10 fold), KA (3-6 fold) and 3-HK (15-17 fold) in the maternal placenta, fetal plasma and brain. In contrast, administration of KA to the pregnant dams increased KA levels in placenta and plasma, but not the fetal plasma or brain. Interestingly, whereas a 7-day treatment with systemic KA in rats led to increased KA concentrations in both plasma and CSF but not in the other metabolites, acute KA administration did alter both TRP and several KP metabolite serum levels (68, 69).

In rats subjected to chronically unpredicted mild stress during 5 weeks, significant increases in KYN were observed in the colon, as well as in the cortex and hippocampus. Additionally, KA levels were increased in colon, whereas a decrease was seen in the cortex and hippocampus. Colonic KYN was significantly correlated with hippocampal KYN (r=.6154; p=.0066), KA (r=−.5787; p=.0119) and 3-HK (r=.5050; p=.0325) and negatively correlated to cortical KA (r=-.6717; p=.0023) (70).

Of note, kynurenine metabolite production seems to vary between species, e.g. between rats and gerbils (71), between mouse and human brain (29), so findings from animal models cannot be generalized to the human brain.

### 3.2 Psychiatric Research

Strong correlations were found between CSF and peripheral measures of KYN in MDD (n=75; r=.61; p<.001 (17) (n=72; r=.60; p=<.0001) (52). Peripheral TRP concentrations, however, did not correlate with central assessments (17, 51, 52), although it should be noted that these studies did not measure free TRP. Notably, the serotonin branch utilizes 10% of the blood-based TRP but on average half of the cerebral TRP, which may partially explain its low correlations with central concentrations. In contrast, peripheral assessments of KA (KAT-branch) did not correlate with CSF measures of the same metabolite in a large sample of bipolar patients (n=163) (20). Plasma concentrations of downstream metabolites of the KMO-branch QUINO and AA also showed a moderate concordance with CSF values in MDD (n=72; r=.55, resp. r=.47; all p-values <.0001) (52).

Although peripheral and central kynurenines have not been compared directly yet in schizophrenic patients, a recent meta-analysis showed that schizophrenia is associated with lower plasma KYN levels but higher CSF KYN and KA (72), which may suggest that peripheral and central KYN are not necessarily correlated in psychotic illness.

However, with only 4 psychiatric studies available, the currently available data do not allow calculation of quantitative meta-analytic summary statistics.

### 3.3 Non-Psychiatric Research

Similar to animal studies and in MDD, both total and free TRP correlation mostly show discordance in studies with healthy participants. Peripheral and central levels of kynurenine metabolites were compared in healthy participants (n=27) either after an intense physical activity or after a 4-week training program. At baseline, PICO levels were strongly correlated between plasma and CSF (r=.93; n=27; p<.0001), whereas a weak correlation was seen for the other kynurenine metabolites. After exercise, however, discordance ensued for KYN (r=-.22; NS), QUINO (r=-.09; NS) and KYNA (r=-.05; NS) (61).

In line with the results in MDD, peripheral KYN levels showed a moderate-to-strong concordances with CSF values in HIV patients (n=79; r=.65; p<.0001) (62), Alzheimer patients (n=20; r=.70; p<.001) (65), hepatitis C (n=27; r=.46; p<.01) (63) and Parkinson patients (n=26; r=.46; p=.03) (64), whereas both free and total peripheral TRP measures again showed conflicting results (free TRP: 3 of 6 studies show discordance; total TRP: 3 of 5 studies show discordance; not specified TRP: 3 of 4 studies show discordance) (53–60, 62, 63).

Peripheral 3-HK assessments also tend to reflect central concentrations of the same metabolite, albeit more modestly, as plasma and CSF 3-HK concentrations correlated weakly in Alzheimer’s (r=.33; p=.044) (65) and moderately in Parkinson’s disease (r=.51; p=.02) (64).

Further downstream the KMO-branch, QUINO and KA were most frequently studied. Blood and CSF assessments of microglia-based metabolites (AA, 3-HK, PICO, QUINO) equally tended to intercorrelate irrespective of the underlying illness (see **Table 1**), with moderate-to-strong concordances for QUINO (r-values ranging from.43 to.57; all p-values below.001) (62, 63, 66) and weak-to-moderate for 3-HK (r-values ranging between.33 and.51; all p-values <.05) (64, 65). Although based on a singly study, plasma AA and PICO showed a high (n=20; r=.63; p<.001) and moderate (n=20; r=.54; p<.001) concordance with CSF levels in Alzheimer patients (65). However, it should be noted that all investigated patient groups had hepatitis or neurological or HIV-related pathology, which may not be reflective of the concentrations and correlations found in psychiatric patients. Serum QUINO in HIV patients for example (73) ranged between 200-8000 nM/L, whereas that in schizophrenia and MDD more modestly ranged from 200 to 600 nM/L (74–76).

In line with findings in BD, KA measures in blood did not mirror central values in patients with Parkinson’s disease and healthy volunteers, although this discordance could be explained by the use of a peripheral aromatic amino acid decarboxylase inhibitor along with L-dopa which inhibit both KAT and KYNY enzymes in the periphery (20, 64).

In conclusion, human studies support several findings from preclinical samples, namely positive correlation coefficients between peripheral and central KYN and 3-HK levels, in contrast to TRP and KA. Free TRP has been preferred by several researchers over total TRP to correlate with CSF values (54, 56, 59, 77), although this could not be confirmed with the current results. Furthermore, hepatitis and HIV patients and immune stimulated rodents showed high inter-tissue correlation values of QUINO, possibly caused by inflammation in both compartments. However, further studies are necessary to confirm these interpretations.

## 4 Link Between Peripheral KP Markers and Endo-phenotypical Markers of Psychiatric Illness

As a secondary objective of this review, we aimed to develop a better understanding of the relationships between peripheral KP markers and several relevant clinical features such as symptom severity and treatment response.

### 4.1 Correlations Between Peripheral KP Markers and Clinical Symptomatology in Psychiatric Illness

An increased KYN/TRP ratio has been associated with higher depression severity scores in MDD patients (78, 79), with the presence of suicidality (80) and with manic symptomatology (81), irrespective of pharmacological treatments. Increased KYN/TRP has equally been linked to reduced cognitive performance in schizophrenia, MDD, panic disorder and aging (17, 76) (**Table 2**). In contrast, symptom severity in depression did not correlate with free or total plasma TRP levels (82).

**Table 2:** Effects of KP changes on symptomatology and biomarkers in psychiatric illness.

Peripheral finding	MDD	BD	SCZ
↑ KYN/TRP	↑ depression severity ↑ cognitive symptom severity ↓ frontal glutamate ↓ striatal volume	↑ mania severity ↓ GM/WM integrity	↑ cognitive symptom severity ↓ GM/WM integrity ↓ DLPFC volumes
↓ KA and/or ↑ QUINO	↑ depression severity ↑ lifetime MDD episodes ↑ ketamine response ↑memory impairment ↓ connectivity ↓ WM integrity ↓ cortical thickness ↓ hippocampal volume	↑ ketamine response ↑memory impairment ↓ connectivity ↓ WM integrity ↓ cortical thickness ↓ hippocampal volume	↑ negative symptom severity ↑ glial cell activity

MDD, major depressive disorder; BD, bipolar disorder; SCZ, schizophrenia; TRP, tryptophan; KYN, kynurenine; KA, kynurenic acid; QUINO, quinolinic acid; WM, white matter; DLPFC, dorsolateral prefrontal cortex.

Increased peripheral QUINO concentrations have been associated with depressive symptom severity in major depression and postpartum depression and in healthy volunteers receiving an inflammatory challenge (10, 83–85), as have increased AA and decreased KA. Decreased KA has also been associated with negative symptomatology in schizophrenia patients (86), although it is important to note that these low KA concentrations could be attributable to extremely low KYN levels (<200ng/ml instead of normal values around 2µg/ml) possibly caused by food intake which is known to lower KYN values (87). It should be noted that these associations were not consistently present (63, 88).

A shift towards the neurotoxic branch has equally been associated to affect cognition, as increased 3-HK activity was associated with poor memory performance in unipolar depressive and bipolar disorder (74, 89). This is in contrast with the negative correlation between KA and social cognition demonstrated by Huang (90). Although serum 3-HK and XA values were lowered in SCZ and BD compared to healthy controls (HCs), these metabolites did not correlate with symptom severity (91).

### 4.2 Correlations Between Peripheral KP Markers and CNS Physiology in Psychiatric Illness

A higher KYN/TRP ratio has been associated with loss of grey and white matter integrity in bipolar disorder (92) and schizophrenia (93) as well as with lower dorsolateral prefrontal cortex (DLPFC) volumes in schizophrenia (76), lower frontal white matter glutamate levels (93) and reduced striatal volumes in MDD (94).

Low plasma KA concentrations and KA/QUINO ratios have been associated with reduced connectivity, white matter integrity and cortical thickness and hippocampal volume in MDD (95–97) and bipolar disorder (75, 98) as well as increased glial cell activation [as assessed by Positron Emission Tomography using radioligand (18F)-PBR111] in schizophrenia (99). Surprisingly, a positive correlation between neurotoxic QUINO and increased connectivity in MDD was equally shown (95).

A small sample of melancholic depressed adolescents (n=7) showed strong correlations (r>.90) between plasma KYN and 3-HAA with brain choline, a cell membrane turnover biomarker, in the striatum with magnetic resonance spectroscopy (MRS) (100). Please find the summary of this paragraph in **Table 2**.

### 4.3 Link Between Peripheral KP Markers and Treatment Response in Psychiatric Illness

In mood disorders, antidepressant treatment with an selective serotonin reuptake inhibitor (SSRI) (48), ketamine (48, 101), electroconvulsive therapy (ECT) (79, 102) or real time functional magnetic resonance imaging (RT fMRI) neurofeedback training (103) typically have similar effects on the KP pathway as evidenced by overall lowering of QUINO and 3-HK levels (typically thought to be more neurotoxic in nature), as well as increases in KA, which is seen as neuroprotective. These KP changes, as well as baseline high QUINO and low KA levels were predictive for treatment response or remission (48, 101, 104). Whereas plasma TRP levels did not predict treatment response on lithium nor amitriptyline, a subnormal ratio of TRP to other amino acids competing with the LAT1 transporter predicted better outcome in depressed individuals (105). Moreover, antidepressant treatment has been shown to decrease IFN-g expression as well as IDO activity in the brain and in peripheral blood mononuclear cells (PBMCs) (106), leading to reduced overall activation of the kynurenine pathway, mirrored by reduced KYN levels after antidepressant treatment (48). It remains to be investigated whether the antidepressant effect of these treatments is mediated by their impact on kynurenine pathway dynamics. Nonetheless, these results suggest that KP abnormalities may be useful as predictive biomarkers for treatment response.

Antipsychotics have equally shown KP modulating effects in schizophrenia patients. Lowered TRP and KA (107, 108) and increased 3-HK levels (108) normalized after antipsychotic treatment. Cao et al. reported lower KYN levels in unmedicated schizophrenic patients in their meta-analysis, whereas higher KYN levels existed during and after treatment with antipsychotics (72). This also accords with earlier findings by our group, which showed that decreased levels of QUINO and 3-HK in unmedicated psychotic patients tend to normalize after antipsychotic treatment (86).

## 5 Peripheral and Central Factors Influencing Kynurenine Metabolite Concentrations

The following sections describe the bioavailability and blood brain barrier transport of KP metabolites in normal and immune-activated conditions, which is often the case in psychiatric illness.

### 5.1 Mechanisms of Entrance of Tryptophan and Kynurenines Into the Brain

The blood-brain barrier (BBB) separates the central nervous system from peripheral circulation and regulates the exchange between these two compartments, protecting the brain from harmful or toxic compounds circulating in the blood, while supplying the brain with nutrients (27).

Tryptophan and kynurenine easily pass the BBB, actively transported by the large neutral amino acid transporter (LAT1) in competition with other essential amino acids such as valine, isoleucine, leucine, tyrosine and phenylalanine (109, 110). LAT1 is ubiquitously expressed on both apical and basolateral sides of the endothelial membranes, as well as on neurons, microglia and astrocytes (111, 112). Although relative concentration differences may be present depending on the compound (113, 114), the transporters provide bidirectional transport to maintain equilibrium of amino acid distribution across both sides of the BBB (115). Preclinical research suggests that 60-78% of the cerebral pool of KYN is imported from the periphery (109, 116, 117) and that TRP transport over the BBB declines with older age (45, 118).

In addition to TRP and KYN, 3-HK is also actively transported over the BBB by LAT1, albeit to a much lesser degree (109, 117). Still, animal research shows that the uptake of systemically administered 3-HK was seven- to eight-fold higher in the brain than in other tissues (119), arguing for efficient metabolite transport over the BBB. AA, another precursor for QUINO (**Figure 1**), can also pass the BBB easily, presumably by passive diffusion (117, 120).

By contrast, QUINO, 3-HAA and KA are not actively transported over the BBB, restricting brain uptake to passive diffusion (120), which supposedly is very limited due to these compounds’ polar nature (117). This is confirmed by preclinical studies where systemically administered neurotoxic doses of KA and QUINO had no effect on rats (121), while equal doses of a synthetic KA variant that easily passes the BBB instantly killed all animals (122). As they cross the BBB very poorly, cerebral concentrations of KA and QUINO are therefore considered mainly to derive from local production (117). In contrast, several gerbil studies show subcutaneously infused radiolabeled QUINO made up 50-70% of the QUINO brain pool (116, 123), challenging the notion that passive QUINO diffusion over the BBB is limited. However, it is unclear to what extent these findings are extrapolatable to humans.

Cholesterol and fatty acid composition of cell membranes determines membrane fluidity and as a consequence the efficiency of the transport function (124). Data from several studies suggest that cholesterol disturbances are associated with psychiatric disease, including MDD and schizophrenia (125–128). Also, chronic hypertension and insulin seem to facilitate TRP brain uptake in rats (129, 130). However, the role of these factors in altered BBB permeability in psychiatric patients needs further clarification.

### 5.2 Bioavailability and Transport of Kynurenine Metabolites

As TRP, KYN and KA are known to be loosely bound to human serum albumin (HSA), these compounds first need to be stripped off of HSA in order to be transported to the central nervous system (131–134). In fact, 80-95% of plasma TRP is bound to human serum albumin (HSA), leaving only a small percentage as free TRP (114, 135). Therefore, factors influencing the albumin concentration or interacting with the binding sites have a major impact on the bound/unbound ratio and, consequently, on transportation over the BBB (136, 137). For example, low HSA may occur during liver and kidney disease, prolonged inadequate food intake and in a pro-inflammatory state. Lower TRP in turn affects albumin synthesis (135, 138). Moreover, fatty acids and several drugs, such as salicylates, are able to displace TRP from its binding site, although these mechanisms are difficult to evaluate *in vivo* (135, 139, 140).

Additionally, it is important to bear in mind that KP enzyme expression and/or activity is changed in various diseases [for review, see (26)].

### 5.3 Peripheral-Central Neuroimmune Crosstalk and the Effect on the Kynurenine Pathway

Although the brain is considered as an immune-privileged organ since tissue grafts survive when implanted into the CNS parenchyma, the BBB has shown to be permeable to inflammatory proteins and cells under inflammatory conditions, which are able to activate immune responses in the CNS. Several mechanisms may result in crosstalk between the peripheral and central immune system, influencing the functional link between the peripheral and central KP metabolism.

Pro-inflammatory cytokines, which activate the kynurenine pathway both peripherally and centrally, pass the BBB easily through cytokine-specific transporters and circumventricular organs (CVOs) (141), the latter being highly permeable and isolated brain areas characterized by efficient neurohumoral exchange (142). However, the complex interactions between the peripheral and central immune system need further clarification. Cytokine-stimulated activation of the kynurenine pathway in the brain may be mirrored in peripheral tissue, and theoretically peripheral assessments of metabolites such as QUINO or KA could indirectly reflect the situation in brain tissue. During immune activation in the CNS, over 98% of brain-located KYN and QUINO could derive from local production (116). However, Guillemin and colleagues (143) demonstrated that human monocytes and monocyte-derived macrophages can produce up to 19 times more QUINO than activated microglia (143). This is also in line with findings of Espey (32), who showed that synthesis of QUINO by microglia in epilepsy patients was approximately 15% of that produced by monocyte-derived macrophages retrieved from brain tissue. In acute liver failure, an 11-fold increase in QUINO plasma concentrations was mirrored by 1-4-fold elevations in postmortem cerebral tissue, again arguing for more potent QUINO production capacities in peripheral tissue (144). In this line, QUINO concentrations over a range of pathological conditions are systematically higher in blood compared to the CNS with blood/CSF ratios of 14:1 in humans, 19:1 in rodents and up to 52:1 in nonhuman primates, [for review, see (145)]. This can also have relevance to the CNS, as infiltrating activated macrophages could be the most potent QUINO source during brain inflammation (143).

Chronic inflammation leads to an enhanced release of pro-inflammatory cytokines and other components that may alter the microvascular permeability, resulting in a so-called ‘leaky’ blood-brain barrier, which is associated with increased permeability for activated monocytes that may migrate to brain tissue, thus exacerbating neuroinflammation (27).

BBB integrity was shown to be affected in 14-29% of treatment resistant patients with mood and psychotic spectrum disorders (146) and was suggested to be associated with negative symptoms in schizophrenia (147). However, very few psychiatric studies focused on differentiating resident microglia from blood-derived macrophages that migrated to the brain, so little is known about a potentially changed macrophage presence in psychiatric brain tissue and their impact on neuroinflammatory abnormalities. Nonetheless, an invasion of macrophages in brain tissue in 40% of schizophrenic patients in a high inflammatory state as found by Cai and colleagues (148), could alter local QUINO concentrations drastically, given the previously mentioned superior ability of macrophages to produce QUINO compared to microglia. Importantly, QUINO increases result in astrocytic apoptosis, which may further impact BBB integrity (149).

As for KA, early results in an animal model may suggest that (supraphysiological) increased levels of peripheral KA could alter BBB permeability in itself, and in this way penetrate the BBB to reach the brain (150, 151). However, the relevance of these data to humans in general and to KP metabolite concentrations reached in psychiatric illnesses specifically is not clear.

The CNS in turn keeps the peripheral immune activity in check by a number of neuronal control mechanisms. First, although both physiological and psychological stress activates the paraventricular nucleus in order to adapt rapidly to threats of homeostasis, the hypothalamo-pituitary-adrenocortical (HPA) stress response has self-regulating abilities through glucocorticoid negative feedback loops (152, 153). Second, stress-induced cortisol activates multiple physiological reactions, including the induction of TDO. On the other hand, acute cortisol release has anti-inflammatory effects, as it leads to the production of anti-inflammatory cytokines (154). However, persistent elevations of cortisol downregulate the expression of the glucocorticoid receptor which results in glucocorticoid resistance, leading to a pro-inflammatory state as evidenced by elevated IL-6 and TNF-a levels (155).

Interestingly, increased cortisol or treatment with dexamethasone has been associated with low TRP plasma levels in treatment resistant schizophrenics (156) and MDD (157–159).

## 6 Discussion

Disturbances in the KP are thought to be involved in the pathophysiology of several psychiatric illnesses, such as psychotic and mood disorders. Whereas these abnormalities are easily measured in plasma/serum, empirical evidence of BBB transportation dynamics of the different metabolites under physiological and pathological conditions is limited. The general consensus has been that TRP, KYN and maybe 3-HK easily cross the BBB whereas other downstream metabolites (QUINO, 3-HAA, AA, KA) do not. Nonetheless, this theory has been based on a single study (117) that investigated BBB KP metabolite transport in rats. Evidently, findings in rodents are not always extrapolatable to humans. Kynurenine pathway enzymes might be more active in the brain of higher species (160) and interspecies differences in the KP have been demonstrated (71, 117). The present review was designed to summarize the available correlation coefficients between peripheral and central kynurenine metabolite concentrations.

In clinical studies, KYN and, to a lesser degree, 3-HK, correlate well between blood and CSF samples, irrespective of underlying diagnosis. This is unsurprising, as both metabolites are actively transported over the BBB. However, although TRP equally passes the BBB easily, CSF and peripheral samples taken from the same individual do not always correlate well in human studies for both free and total TRP, especially with regard to more recent studies. High TRP-binding to blood albumin may explain low correlations to central TRP levels. More than 90% of the peripheral TRP is metabolized into KYN, while brain TRP is equally divided over the serotonergic and the kynurenine pathway (69) Additionally, IDO is more represented in the brain, whereas TDO is mainly responsible for metabolization in the periphery; this may contribute to differential levels between CNS and blood. As TRP is an essential amino acid, active transport into the brain may alter the ratio of these molecules between CSF/brain and blood. Downstream metabolites in both branches of the pathway cannot easily pass the BBB due to a lack of active transportation. Yet despite relying solely on passive diffusion, peripheral and central concentrations of QUINO and the precursor AA have shown correlations of moderate strength in neurological (Parkinson’s disease, Alzheimer’s disease) and infectious disorders (HIV, hepatitis C). In psychiatric disorders however, there generally is a lack of available evidence on CSF concentrations of kynurenine metabolites. Interestingly, out of all KP compounds, peripheral KA levels seem to be the least predictive and even diametrically opposed to those in the CSF of depressed and bipolar patients.

**Figure 3:**
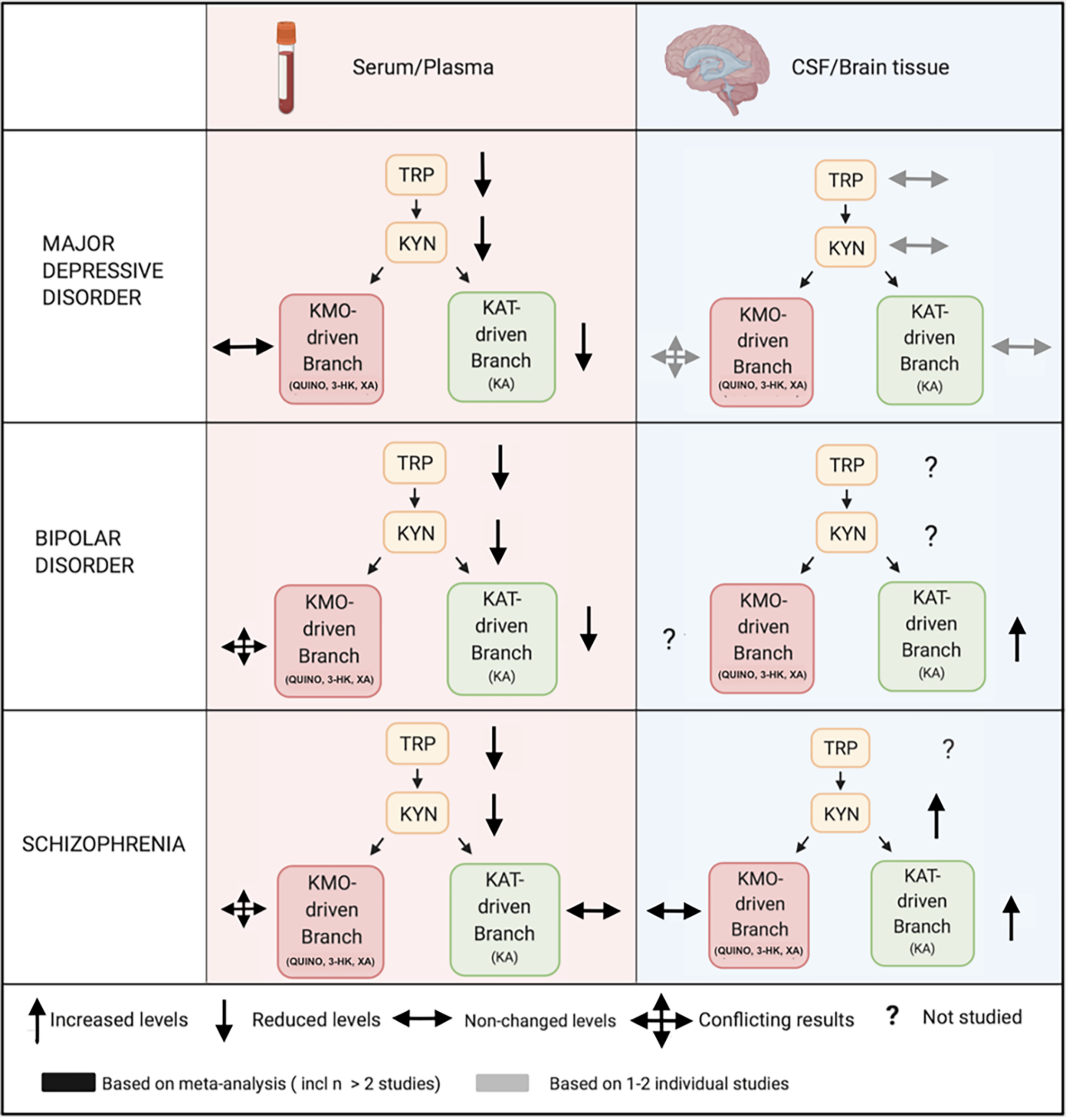
Comparing peripheral (serum/plasma) and central (CSF/brain tissue) kynurenine pathway findings in major psychiatric disorders. Legend: In mood disorders, peripheral studies demonstrate decreased measures of blood TRP, KYN and a downregulation of the KAT-driven branch in the periphery, which is possibly explained by a decreased availability of TRP to the KP. Central studies investigating cerebrospinal fluid (CSF) or brain tissue are less conclusive, but are very limited. In schizophrenia, peripheral findings are much less clear downstream with very conflicting results. Central studies in schizophrenia, also limited and mainly based on CSF research on KA, equally suggest an activation of the pathway, reflected by KYN increases, accompanied by a shift towards the astrocyte-derived branch. KP, kynurenine pathway; 3-HK, 3-hydroxy-kynurenine; KA, kynurenic acid; KYN, kynurenine; QUINO, quinolinic acid; TRP, tryptophan; XA, xanthurenic acid; KMO, kynurenine 3-monooxygenase; KAT, kynurenine aminotransferase.

Overall, recent meta-analyses and individual psychiatric studies investigating KP metabolites in either peripheral or central samples have shown divergent results across both sides of the BBB (**Figure 3**). Several reasons can be proposed for these discrepancies. It is to be considered that central and peripheral aberrations may reflect different processes in the human body. Under physiological circumstances for example, intense physical exercise causes transient changes in the KP both in the periphery and centrally (61, 161, 162). KP metabolization in tissue macrophages, PBMCs and other immune cells contribute substantially to peripheral concentrations, whereas central levels of downstream metabolites are mostly determined by the lower enzymatic activity in astrocytes and microglia. Moreover, somatic comorbidities such as autoimmune illnesses, metabolic syndrome, an altered microbiome and hepatic or renal dysfunction (163, 164) may impact peripheral and central KP metabolite concentrations. Even though stress and pro-inflammatory cytokines (IFN-g, TNF-a) equally activate the pathway in peripheral and central tissue (123), it is possible that the correlation between peripheral and CNS KP findings depends on the type and level of inflammation. While high-level inflammation and/or BBB disintegrity may lead to parallel changes in CNS and blood KP (**Table 1**), this is not necessarily the case in the chronic mild inflammatory conditions found in psychiatric illness (**Figure 3**). The observed positive correlations in blood-CSF QUINO concentrations in MDD, hepatitis and HIV, but not in healthy volunteers, could be attributable to the inflammatory conditions in these illnesses damaging the BBB. On the other hand, peripheral findings in psychiatric illness do represent valuable biomarkers, associated with symptom severity and treatment response, as well as other core biological features of the disorder as discussed in section 6. This corroborates the notion of psychiatric illnesses as ‘whole-body disorders’ rather than brain disorders (165).

Psychiatric diagnoses are typically based on clinical phenotypes which are now widely accepted to represent a heterogeneous group of biotypes. Several biological dysregulations (immune, neuroendocrine, metabolic,…) are therefore nonspecific, overlapping between diagnostic categories. Kynurenine abnormalities may thus vary over studies depending on the representation of the different biotypes in these studies. Symptomatic state (14), medication status and use of illicit substances - in particular THC - (86, 108, 166) may also impact KP alterations, further adding to the variability. The complex interaction between KP and the dimensional aspects of different psychiatric syndromes needs further scrutiny. An increased KYN/TRP ratio as well as low KA and/or high QUINO emerge as true transdiagnostic blood-based trait markers across the three major psychiatric disorders. A similarly strong overlap has recently been demonstrated in the polygenic risk factors related to these disorders (167) (see **Table 2**).

Methodological issues should also be considered. Study sample sizes have systematically been small, especially in those investigating central KP metabolites. A recent meta-analysis (19) demonstrated that schizophrenia-related KYN findings in CSF were based on a total of 60 patients over 3 studies, and KA on a total of 148 patients over 4 studies. Although the role of QUINO in the pathophysiology of schizophrenia has been a favored and frequently repeated hypothesis in many opinion papers and reviews, no study has actually investigated QUINO in the CSF of schizophrenia patients to date. Similarly, the KP was investigated in the CSF of below 100 MDD patients (17–19). Postmortem studies equally tend to include smaller sample sizes (168–170). The fact that data on microglia-driven metabolites appear more mixed than KA levels, may simply result from the fact that these metabolites have hardly been investigated. Another methodological issue is that some metabolites (e.g. QUINO) are present in very low ranges (200-600 nM/L) and QUINO findings over studies differ with factors up to one million, which may reflect bioanalytical inaccuracies related to the assays’ lower detection limits that are nonetheless rarely acknowledged in these papers (171). In this line, the smaller ranges of KP metabolite alterations found in psychiatric illnesses (75, 76) may need higher sample sizes in order to achieve sufficient statistical power compared to studies investigating neurological and infectious illnesses, which are accompanied by high-grade systemic responses and more profound blood-brain barrier disintegrety (63, 65, 73). Another issue is that CSF findings may not reflect brain KP metabolism *in situ*, especially in the context of a ‘leaky’ brain, as CSF may be more representative of KP enzyme activity in periventricular macrophages rather than parenchymal glial cells. Moreover, the total volume of the obtained CSF and the exact intervertebral height have a significant impact on protein concentration as the concentration decreases when descending along the vertebral column (172).

Finally, KP enzyme activity is typically estimated using metabolite ratios. For example, TDO/IDO activity is typically calculated from KYN/TRP ratios. Actual assessment of enzyme activity (e.g. in isolated cell types such as PBMCs) or genetic expression of these enzymes may be more relevant and representative.

In conclusion, KYN and 3-HK measured in plasma or serum seem to reflect their concentrations in brain tissue, but this relationship is less clear in TRP and more downstream metabolites of the KP. Even if peripheral concentrations do not correlate with central measures in psychiatric illness, they are not necessarily without merit as relevant biomarkers of phenotypical features and treatment response. Nonetheless, many potential confounders may contribute to diverging central and peripheral assessments, and more fundamental research is needed to clarify these issues. Future studies should investigate 1) to what extent KP metabolites pass BBB in humans (e.g. by use of radioactive labelling), 2) whether CSF concentrations reflect *in situ* brain abnormalities and to what extent these KP abnormalities are systemic or region-dependent in the brain, 3) how well CSF and blood concentrations of microglial branch metabolites intercorrelate in mood and psychotic disorders and 4) what the impact is of medication, symptom status and illness phase on KP abnormalities. Finally, we strongly recommend future studies investigating the KP in psychiatric illness to assess (at least) the following metabolites: TRP, KYN, QUINO, 3-HK and KA.

## Data Availability Statement

The original contributions presented in the study are included in the article. Further inquiries can be directed to the corresponding author.

## Author Contributions

KS, LP, VC, and MM equally contributed to the study design of this review. KS and MM performed the literature search, interpreted the data and wrote the manuscript. LP, VC, and RV profoundly ameliorated the manuscript by adding important intellectual content. All authors contributed to the article and approved the submitted version.

## Conflict of Interest

The authors declare that the research was conducted in the absence of any commercial or financial relationships that could be construed as a potential conflict of interest.

## Publisher’s Note

All claims expressed in this article are solely those of the authors and do not necessarily represent those of their affiliated organizations, or those of the publisher, the editors and the reviewers. Any product that may be evaluated in this article, or claim that may be made by its manufacturer, is not guaranteed or endorsed by the publisher.
